# Conservative Non-Surgical Management of Horizontal Root-Fractured Maxillary Incisors in a Young Male with Angle Class II, Division 2, Malocclusion

**DOI:** 10.3390/dj9050055

**Published:** 2021-05-12

**Authors:** Roberto Biagi, Giulia Bardini, Giuseppe Guidazzi, Enrico Spinas

**Affiliations:** 1 Department of Biomedical, Surgical and Dental Sciences, School of Dentistry, University of Milan, Via Della Commenda 10, 20122 Milan, Italy; 2 Department of Conservative Dentistry and Endodontics, University of Cagliari, Via Ospedale 40, 09124 Cagliari, Italy; supergiu.gb@gmail.com; 3 Private Practice of Orthodontics, Viale Abruzzi 80, 20131 Milan, Italy; studioguidazzi@libero.it; 4 Department of Surgical Sciences, Sports Dental Research Center, University of Cagliari, Via Ospedale 40, 09124 Cagliari, Italy; enricospinas@tiscali.it

**Keywords:** trauma, dental injury, horizontal root fracture, splinting, orthodontics

## Abstract

Horizontal root fractures are a rare emergency in a dental office. The injury involves periodontal ligament, cementum, dentine and pulp. The healing is influenced by the location of the root fracture, the displacement of the fragments and the status of the pulp. This report presents a clinical case of horizontal fractures to both maxillary central incisors due to an act of violence. The type of occlusion has avoided a severe diastasis of the coronal parts with a subsequent damage to the pulp and periodontum. The fractures were treated with an orthodontic splint without any further therapy and hard tissue healing was observed. A careful diagnosis and well-timed treatment planning usually allow a cost-efficient and biologically-oriented therapy with a favorable outcome.

## 1. Introduction

Root fractures represent up to 7% of traumatic injuries to permanent teeth, mainly to central and lateral maxillary incisors. Root fractures normally result from a frontal impact that causes combined injuries to pulp, dentine, cementum and to periodontal ligament and alveolar bone, and are broadly divided in horizontal and vertical root fractures [1]. The plane of fracture is dictated by the shearing stress zone. The most common root fractures are horizontal type and they may occur at the middle third (57%), followed by apical third (34%) and cervical third of the root (9%) [2]. The modalities of repair after root fractures are healing with calcified tissue or with interposition of connective tissue with or without bone; however, non-healing with interposition of granulation tissue is also possible. The stage of root development, the location of the root fracture, the extent of dislocation and repositioning, the type of splinting and the use of antibiotics are important factors for long term survival rates [1,3,4].

The case here described regards a 20-year-old male who suffered the root fractures of teeth 1.1 and 2.1 following physical violence, and it shows how this type of occlusion has avoided a severe diastasis of the coronal parts, thus promoting a favorable prognosis.

## 2. Case Presentation

**Figure 1:**
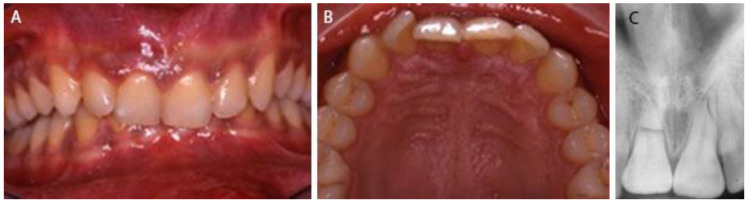
At the time of traumatic injury, (**A**) buccal clinical view, (**B**) occlusal clinical view and (**C**) periapical X-ray.

A healthy 20-year-old male was referred to the dental office for pain during his breakfast, probably due to traumatic injuries to the teeth following an affray occurring the night before. On clinical examination both maxillary central incisors were subluxated and attached gingival of the right one was tender to palpation. The lateral maxillary incisors were not affected by the trauma and were tested vital. Radiographic examination revealed a horizontal fracture of the right incisor at the middle of the root and a second one of the left incisor at the coronal two third portion of the root (Figure 1). No preoperative cone-beam computed tomography was taken due to limitations of emergency. Pulp testing of both incisors was uncertain.

**Figure 2:**
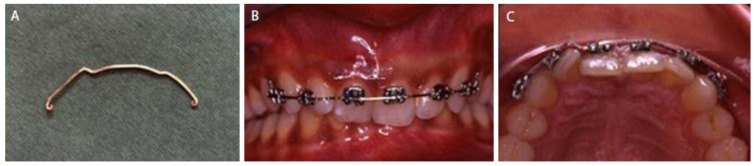
At the time of orthodontic splinting, (**A**) 0.017 × 0.025-in orthodontic stainless-steel wire splint with first order bends, (**B**) buccal clinical view and (**C**) occlusal clinical view.

After the informed consent, under local anesthesia, 0.022 × 0.028-in Roth prescription brackets were bonded to the labial surfaces of the right and left incisors and cuspids. A splint was made with 0.017 × 0.025-in orthodontic stainless-steel wire that was given first order bends to retain the forward passive positioning of the incisors during splinting (Figure 2).

**Figure 3:**
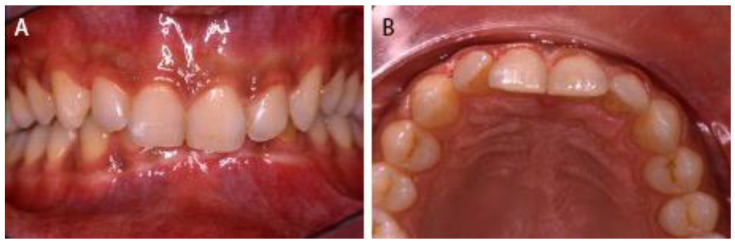
At the time of splint removal (after 12 weeks), (**A**) buccal clinical view and (**B**) occlusal clinical view.

A periapical radiograph was obtained to confirm the proper positioning of the coronal fragments. The patient received instruction about soft diet and oral hygiene: brushing his teeth with a soft toothbrush after each meal and rinsing his mouth twice a day for one week with 0.20% chlorhexidine. Systemic antibiotic (amoxicillin 2 g each day for one week) and analgesic medicament on demand were prescribed. Recall visits were planned according to the International Association of Dental Traumatology guidelines. The splint was removed after 12 weeks (Figure 3). The healing of both incisors was achieved with interposition of hard tissue. No sign of pulpal necrosis was observed, and the teeth showed positive response to pulp testing.

**Figure 4:**
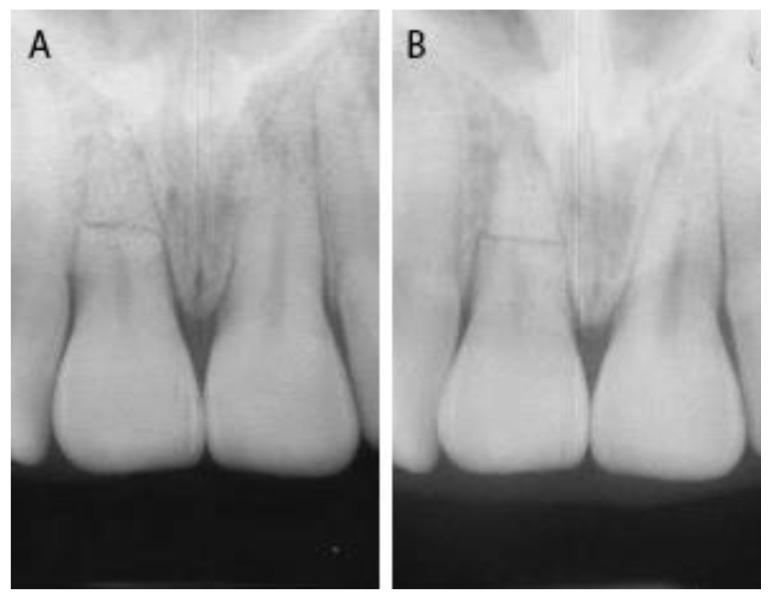
Follow-up, (**A**) periapical X-ray at three-month recall visit after splint removal and (**B**) periapical X-ray at twelve-month recall visit after splint removal.

At 3-month and 12-month recall visits after the splint removal, the patient reported no discomfort and there was no sign of pathology (Figure 4).

## 3. Discussion

The increase in the severity of violence among young people, most frequently in the age group 21–25 years, is an important social problem: up to 6.6% traumatic dental injuries are due to physical violence [5,6].

In this clinical case, trauma caused two simple horizontal root fractures: the first at the middle third of the root of 1.1 and the second one at the apical third of the root of 2.1.

The prognosis of a tooth with root fracture depends on the stage of root growth, the extent of the fracture line, the dislocation of the fragments, the pulp tissue status, the occlusion, and the general health condition of the patient. The frequency of pulp necrosis following root fractures is higher in mature teeth than in immature ones: widely patent apice and short pulp length facilitate pulpal revascularization [7]. Since the apical fragment remains essentially injured, the degree of coronal fragment displacement is an important conditioning factor in fracture healing, and it can be considered as a luxation injury [8], with a consequent trauma to the periodontal ligament and neurovascular supply of the coronal pulp. Even if the radiograph suggests fragmentation of the pulp, the pulp is not necessarily severed, in which case root canal treatment can be avoided [9].

In this report the two incisors completed their development. The retroclination of the upper central incisors and their increased overbite (Angle class II, division 2, malocclusion) avoided a severe diastasis of the fragments, with subsequent damage to the pulp and periodontum.

Ideal repositioning of the coronal fragment optimizes the healing because it reduces the distance between the fragments and the risk of invasion of bacteria through the coagulum in the fracture line; if the fragments are approximated soon after the fracture, there is a good chance that no endodontic treatment is necessary. In this report, the absence of dislocation of the coronal part of the teeth had a significant impact upon healing.

Splinting is significantly associated with the healing outcome too, and many splinting types have been proposed over the years. It has been suggested that a splint is best placed within 24 h [10]. In this case the teeth were stabilized with an orthodontic splint about 12 h after the trauma. The brackets were positioned to minimize any possible occlusal trauma and first order bends were given to retain the forward passive positioning of the incisors during splinting, keeping in mind that with this technique caution must be exercised not to apply any orthodontic forces to the splinted teeth, as not to develop stress that disturbs their healing phase. The splint was retained for 12 weeks. A rigid splint implies a restriction of mobility between the fragments with a consequent reduced circulation and possible disturbance of natural healing events. Additionally, the forces exerted to remove a rigid splint may disturb the periodontal healing of the injured teeth and cause damage to the enamel [11,12]. Currently, the use of a flexible splint is supported to allow physiological movements of the traumatized teeth, and therefore promote hard tissue healing, for 4 weeks, when the fracture is localized at the apical or the middle third of the root [13,14]. Splinting duration seems not to be a significant factor if related to healing outcome [15,16]. Finally, no difference in the frequency of healing between splinted and non-splinted teeth was observed when there was no dislocation of the coronal fragment [11].

Optimal oral hygiene and the use of a chlorhexidine mouth rinse may prevent bacterial contamination through the gingival pocket and help induce healing [17]; for this reason, 0.20% chlorhexidine twice a day for one week was prescribed.

Antibiotics should be only prescribed in case of severe injury with infected wounds. An increase of pulp necrosis and an interference with hard tissue healing after antibiotic therapy when root fractures occur has been reported in literature. Further studies are necessary to clarify this controversial aspect [18,19]. However, in this case amoxicillin 2 g each day for one week was prescribed.

The follow-up period for this report was not very long. After 3–6 months it becomes possible to determine in a reliable manner the fracture-healing type [20]; for this reason, a 12-month recall visit after the removal of the splint demonstrated the positive outcome: healing with calcified tissue across the fractures and vitality of the teeth.

## 4. Conclusions

Incorrect treatment of root fractures, such as their unnecessary endodontic treatment, is often performed; moreover, root fractures are considered by many authors as main candidates for replacements with implants. The long-term prognosis of root-fractured teeth seems to be closely related to the position of the fracture line and to the type of healing. Horizontal fractures to the apical or the middle third of the root with no or minimal dislocation of the fragments usually have a favorable outcome.

## Data Availability

The data presented in this study are available in this article.
